# Effectiveness of Home Bleaching Treatment after Resin Infiltrant Application

**DOI:** 10.3290/j.ohpd.a44691

**Published:** 2020-07-04

**Authors:** Rafael Santos Rocha, Maurício Yugo de Souza, Laura Célia Fernandes Meirelles, Carolina Gigli Torres Scarense, Michele Baffi Diniz, Taciana Marco Ferraz Caneppele, Eduardo Bresciani

**Affiliations:** a PhD Student, Department of Restorative Dentistry, Institute of Science and Technology, São Paulo State University (UNESP), São José dos Campos, SP, Brazil. Laboratory study phase, data discussion, wrote the manuscript.; b Dentist, Department of Restorative Dentistry, São Paulo State University (UNESP), Institute of Science and Technology, São José dos Campos, SP, Brazil. Data discussion, wrote the manuscript.; c Dentist, Department of Restorative Dentistry, São Paulo State University (UNESP), Institute of Science and Technology, São José dos Campos, SP, Brazil. Laboratory study phase.; d Assistant Professor, Paediatric Dentistry, Institute of Dentistry, Cruzeiro do Sul University (UNICSUL), São Paulo, SP, Brazil. Data discussion.; e Associate Professor, Department of Restorative Dentistry, São Paulo State University (UNESP), Institute of Science and Technology, São José dos Campos, SP, Brazil. Data discussion, wrote the manuscript.; f Associate Professor, Department of Restorative Dentistry, São Paulo State University (UNESP), Institute of Science and Technology, São José dos Campos, SP, Brazil. Study design, statistical analysis, data discussion, wrote the manuscript.

**Keywords:** colour, dental caries, dental white spots, aesthetics, tooth bleaching

## Abstract

**Purpose:**

Resin infiltration may be a barrier for bleaching gels. The aim of this study was to compare dental bleaching effectiveness using low-concentration gels on heavily or mildly stained teeth that were or were not treated with resin infiltration agents.

**Materials and Methods:**

Forty bovine enamel surfaces were submitted to demineralisation, after which two staining protocols were performed. Twenty specimens were immersed in a staining broth for 24 h (Lab 1) or 7 days (Lab 2). Ten specimens of each group received resin infiltrant application following the manufacturer’s recommendation. All specimens were bleached using 15% carbamide peroxide gel for 14 days (8 h daily). Colour measurement was performed using a reflectance spectrophotometer at three time points: baseline, after staining, and after bleaching. Data (CIEDE00) were analysed using Student’s t-test (p < 0.05).

**Results:**

No statistically significant differences were observed in Lab 1 (p = 0.560). For Lab 2, statistically significant differences were detected (p = 0.031). Once bleaching was achieved to some degree (Lab 2), the resin infiltrant may have behaved as a semipermeable barrier to the carbamide peroxide gels.

**Conclusion:**

Bleaching treatment was effective on mildly pigmented tooth surfaces. On the other hand, in comparison to the control group, the heavily pigmented surfaces bleached less in the presence of the resin infiltrant, possibly due to the lack of free radicals penetrating into the substrate.

Disturbances during enamel development may be related to enamel colour alteration. These enamel defects present different aetiologies based on pre-eruptive conditions (fluorosis, molar-incisor hypomineralisation, traumatic hypocalcification). Moreover, even after tooth eruption, other conditions may affect the enamel surface, such as an imbalance in the demineralisation-remineralisation process resulting from poor hygiene (dental caries).^5^ This condition creates porosities in enamel that appear clinically as white spot lesions (WSL).

The structural alteration in enamel caused by such conditions affects the dental surface. It is reported that the highest incidence of WSL is concentrated in the anterior maxillary teeth,^7^ which leads to undesired aesthetic consequences often reported by patients. If the enamel appearance affects the quality of the patient’s life and if requested by patients, aesthetic treatments might be an alternative.^5^

One of the most common causes for the clinical development of WSL is related to orthodontic treatment, since fixed appliances are bonded onto the enamel surface.^16^ The use of fixed appliances increases the opportunity for bacterial biofilm retention due to the presence of brackets, bands, and other devices bonded on teeth, besides impairing toothbrushing.^30,33^

Biofilm retention around orthodontic devices results in areas of decalcification^16^ known as WSL. WSL usually present a white, opaque appearance^25^ as a consequence of tooth porosity, since the presence of air in pores leads to a different light refraction index.^23,30^ Although the literature reports several protocols for the removal or masking of these lesions, no consensus has been established regarding the ideal protocol.^25^ Among treatment options, resin infiltration has been shown to be a non-invasive and predictable treatment that improves the smile and dental aesthetics,^28^ and can help prevent caries progression or cavitation.^31^

Resin infiltration has been shown to fill intercrystalline spaces. This consequently changes the light refraction in the lesion area, thus leading to an optical aspect more similar to unaffected dental enamel.^32^ It is a less invasive treatment when compared with microabrasive techniques^2^ or aesthetic restorations such as direct veneers.^19^ It is reported that after conditioning with HCl (the first step of the infiltration approach), close to 34 µm of enamel is removed.^22^ In contrast, the microabrasive technique is reported to remove approximately 200 µm of enamel thickness.^20,26^ Differences regarding both treatment protocols are related not only to the removal of dental tissue, but also to the fact that filling the body of the carious lesion with resin is possible only with the infiltration technique.

Besides the strengthened and filled carious lesion,^5^ a side effect of the infiltration technique might be optical adaptation. Considering aesthetics, the main objective of the resin infiltration technique is to remove the whitish aspect of the affected enamel surface,^32^ which is characteristic of carious lesions. It has also been used to treat other problems not related to caries, such as dental fluorosis.^3,27^ In non-severe cases of white spots, teeth lose their whitish characteristic after resin infiltration, leading to a more natural appearance.^3^

Although the results of infiltration treatment are still unpredictable to date, a common clinical concern of patients is what the final colour of their teeth will be after infiltration. This concern is due to the fact that after the removal of WSL and the teeth present a more uniform colour aspect, the treated teeth lose their whitish appearance.^11^ Patients usually perceive a darker enamel surface after the infiltration procedures. It is reported that the resin infiltration technique can increase b* values, resulting in yellowing of the tooth surface,^11^ and this characteristic is clinically perceptible.^21^ Moreover, some authors also suggest that TEG-DMA (component of infiltrating resin matrix) may favour water absorption,^24^ leading to possible colour instability^13^ and consequent staining.^10^

Based on the possible perception of tooth colour change by patients right after resin infiltration, a bleaching treatment might be required. In an earlier study,^27^ the association of bleaching followed by resin infiltration yielded adequate bleaching results. The fact that resin fills into enamel pores^23^ suggests that it may behave as a barrier for the bleaching products to penetrate into dental structures, and the results of the bleaching procedure might be suboptimal. Although bleaching of stained infiltrated layer has been reported,^1^ the literature contains little on the bleaching response after resin infiltration of dental structures.

Thus, the aim of this in vitro study was to compare the efficacy of dental bleaching with low-concentration gels on heavily or mildly stained teeth that were or were not treated with resin infiltration agents. The null hypothesis was that the infiltrating agent does not influence bleaching efficacy.

## Materials and Methods

Ethical approval was obtained from the local Institutional Review Board on animal studies (protocol#06/2019). Forty demineralised bovine enamel specimens were used in the present study. They were randomly divided into four groups according to the staining regimen and infiltrant resin application.

**Fig 1:**
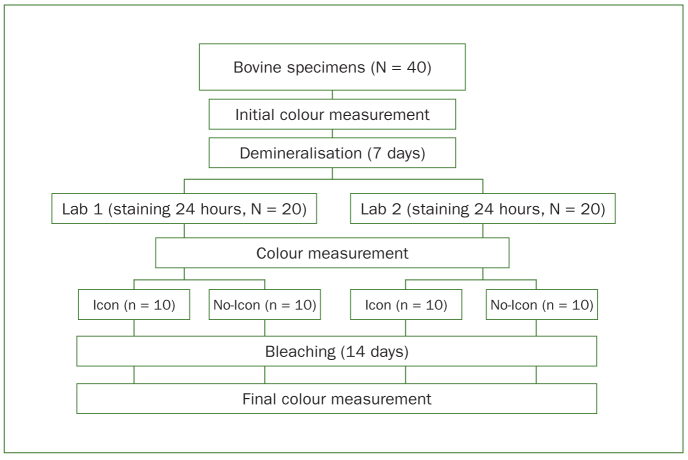
Study design.

The schematic diagram with methodology is presented in Fig 1.

### Sample Preparation

Forty circular anterior bovine dentin/enamel samples were obtained from the buccal surface using a trephine diamond drill with a 6 mm internal diameter. Specimens were initially inserted into the device with the dentin surface facing out. Dentin was then smoothed flat in a circular polishing machine (DP-10, Panambra; São Paulo, SP, Brazil) at 300 rpm with sandpaper P1200. The specimens were removed from the device and repositioned with the enamel surface facing out. The enamel surface was smoothed flat with sandpaper P1200 to standardise the thickness at 0.5 mm, and further polished with sequential sandpapers (P1200 and P2400 for 30 s and P4000 for 60 s) in a circular polishing machine.

All specimens were positioned in a silicone mold with the enamel surface facing the bottom of the mold space. The mold was filled with self-curing acrylic resin (Jet Classic; São Paulo, SP, Brazil) resulting in a specimen with only the enamel surface exposed. For polishing, the samples were placed in a metal device with a central perforation 6 mm in diameter and an adjustable depth. Dentin thickness was standardised at 1 mm. The enamel surface was smoothed flat with sandpaper P1200 to standardise the thickness at 0.5 mm, and further polished with sequential sandpapers (P1200 and P2400 for 30 s and P4000 for 60 s) in a circular polishing machine.

### Colour Measurement

The colour measurement was performed at three time points (baseline, after staining, and after bleaching) using a reflectance spectrophotometer (CM 2600d, Konica Minolta; Osaka, Japan), according to the Commission Internationale de l’Eclariage (CIE)L*a*b*.^8^ The device was set to read small samples (SAV) with a 100% ultraviolet light, 2-degree observation angle, and specular reflection. Data were collected using the software SpectroMagic N X (Konica Minolta). The colour value for each datapoint was obtained from the mean of three consecutive readings performed on a standard white background (L: 84.95; a: -0.38; b: 2.93).

Calculations for the CIEDE00 (ΔE00) colour difference formulas were made according to the following equation:^8,29^

ΔE00=[(ΔL’/KLSL)^2^+(ΔC’/KCSC)^2^+(ΔH’/KHSH)^2^+RT(ΔC’/KCSC)ΔH’/KHSH)]^1/2^

where ΔL’, ΔC’, and ΔH’ are the differences in lightness, chroma, and hue before and after aging in CIEDE00, and RT (rotation function) accounts for the interaction between the chroma and hue differences in the blue region. The weighting functions SL, SC, and SH adjust the total colour difference for variation in the location of the colour difference in L*, a*, and b* coordinates. The parametric factors KL, KC, and KH are correction terms for experimental conditions.

### Demineralisation Process

**Fig 2:**
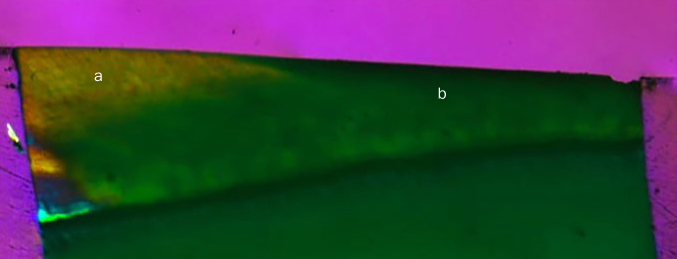
Subsurface lesions under polarised light microscopy. a: unprotected surface with the presence of enamel demineralisation; b: no demineralisation due to surface protection.

Artificial subsurface enamel lesions were produced by immersing samples in a solution, as described by Buskes et al.^6^ The demineralising solution was composed of a 10-M potassium hydroxide buffer solution containing 3 mM CaCl_2_•2H_2_0, 3 mM KH_2_PO_4_, 40.573 mM CH_3_COOH, and 10 ml CH_6_O_6_P_2_, with a final pH = 4.95. The specimens were individually immersed in the solution at 37ºC for 7 days. The total solution volume used was calculated using 2 ml/mm^2^ of the enamel area. This method produced subsurface enamel lesions with a mean depth of 43 µm.^18^ The protocol used for creating subsurface lesions was confirmed by pilot tests under polarised light microscopy (Fig 2, 5X magnification). The yellow region (A) represents the sample area where surface protection was not applied and demineralisation occurred. The green region (B) represents the sample area in which the surface was protected and demineralisation did not occur.

### Staining

All samples were stored in microtubes containing 2 ml of a staining broth: 1.5 g finely ground instant coffee, 1.5 g finely ground instant black tea, 0.33 ml FD & C Red 40 and 0.33 ml FD & C Yellow 5, 41.6 ml red wine, 0.125 g methylparaben, and 0.075 g propylparaben, all dissolved in 0.5 l of water (adapted from Wozniak et al^34^). Half of the specimens (n = 20) were stained for 24 h (Lab 1) and the other half for 7 days (Lab 2). The different staining protocols were performed to test extreme staining conditions and also the conditions usually given in clinical scenarios, with the latter taking the previously determined acceptable ΔE value (ΔE = 2.7)^21^ into consideration. After staining, the specimens were washed with deionised water and a second colour measurement was performed.

### Resin Infiltrant Application

Ten random specimens from each staining protocol received the resin infiltrant (Icon, DMG; Hamburg, Germany) following the manufacturer’s instructions. The enamel surface was etched with Icon-Etch for 2 min, followed by thorough rinsing and drying. Ethanol was applied for improved drying. Two applications of Icon Infiltrant were performed: one application of 3 min and a reapplication of 1 min. Light curing was performed between and at the end of resin applications. The other ten specimens from each group were not resin infiltrated (control groups). All specimens were then immersed in artificial saliva for 24 h.

### Bleaching

Bleaching was performed in all groups using 15% carbamide peroxide gel (Ultradent; South Jordan, UT, USA) on enamel surfaces for 8 h/day for 14 days, following the manufacturer’s guidelines. At the end of bleaching, a third colour measurement was performed.

### Statistical Analysis

The normality of the data was confirmed by the Kolmogorov-Smirnov test. Data were submitted to Student’s t-test for Lab 1 and Lab 2 tests individually (p < 0.05).

## Results

For Lab 1, with the ΔE00 value consistent with clinical scenarios, no statistically significant differences were found for the ICON application (p = 0.560), thus the presence of ICON did not influence the whitening efficacy (Lab 1). On the other hand, for Lab 2, with samples presenting extreme staining, statistically significant differences were detected (p = 0.031), showing that ICON did hinder the whitening efficacy (lower ∆E00). A greater ΔE00 value was observed in the non-ICON group, suggesting a greater bleaching effect (Table 1).

**Table 1:** ΔE value of CIEDE00 for both studies

ICON	ΔE00 – Lab 1	ΔE00 – Lab 2
With	2.525 (± 1.164)^A^	23.800 (± 6.580)^a^
Without	2.263 (± 0.806)^A^	29.473 (± 5.174)^b^

Different superscript letters represent differences for ICON application within each staining condition (Student’s t-test).

## Discussion

The treatment of white spot lesions with resin infiltration has been increasingly employed due to its ability to decrease caries lesion progression and to mask the whitish aspect of WSL.^5^ According to the literature, demineralisation of the enamel intercrystalline spaces occurs along with WSL development.^23^ After demineralisation, porous areas are formed and, when filled with water or air (IR = 1.33 and IR = 1.0, respectively), present a different refraction index (IR) than does healthy dental enamel (IR = 1.62) and are thus seen as white spots.^23^ Due to the low viscosity of the resin infiltrant, this material is able to infiltrate and fill the intercrystalline spaces of the demineralised areas, consequently leading to a change in light refraction. The infiltrating agent presents IR = 1.52, a value that is much closer to healthy enamel’s IR, thus favouring the masking of WSL.^23^

Data from this and previous studies show that the resin infiltration technique may interfere with the tooth’s aesthetic appearance in relation to colour achieved after treatment (ΔE = 3.89 ± 1.22, data not presented). According to Paravina et al,^21^ ΔE > 1.2 was noticeable by at least 50% of observers, while ΔE ≥ 2.7 was considered unacceptable.^21^ Observations from the present study corroborate with Hallgren et al,^11^ who evaluated the colour change of WSL after resin infiltration, detecting a significant colour change (ΔE = 2.0). Based on this information, bleaching treatment might be required by patients after infiltration treatment, which supports the objective of the present study.

Little is known about the influence of bleaching on teeth treated with resin infiltration, considering that ICON resin could behave as a barrier to bleaching gel penetration as a result of the resin’s infiltrating ability (as deep as 0.67 mm).^9^ Based on the results obtained here, the null hypothesis that the infiltrating agent does not influence the bleaching efficacy can be partially accepted. Regarding the staining protocol consistent with clinical situations (Lab 1), there was no statistically significant difference between the test and control groups. However, in terms of extensive staining (Lab 2), a reduced bleaching efficacy was observed for the carbamide peroxide based agents.

Although the literature reports evaluations of the diffusion capacity of bleaching agents,^14,15^ it is known that the bleaching efficacy of carbamide/hydrogen peroxide-based gels relies on the permeability of free radicals into the enamel structure.^4^ After enamel penetration, oxidising free radicals reach the dentin substrate and gradually infiltrate into deeper regions, a fact dependent on the intrinsic characteristics of this substrate.^4^ Thus, sealing the enamel surface might interfere with the penetration and consequent efficacy of bleaching agents. Analogous to studies evaluating bleaching efficacy in patients with orthodontic brackets, it was observed that bleaching gel was not able to effectively change the colour just below the fixed appliance.^12,17^ This may be related to the fact that the bracket and cement might have posed a barrier to the penetration of the bleaching agent.^4,12,17^

In the present study, the resin infiltrant may have acted as a semipermeable physical barrier, leading to reduced enamel surface permeability, an assumption based on the fact that both tests revealed bleaching to some degree. In this study, when the staining protocol simulating clinical staining (fewer pigments within the tooth structure) was employed (Lab 1), the amount of free radicals overcoming the supposed infiltrant barrier may have been able to promote the breakdown of pigment molecules, resulting in tooth bleaching. However, in the presence of extensive pigmentation (Lab 2), bleaching efficacy was reduced by the possibly lower amount of free radicals overcoming the physical barrier of the resin infiltrant, and consequently did not promote the breakdown of all pigment molecules. In the latter scenario, bleaching did not effectively promote colour change when compared to the control group (without ICON). Because of the inferior bleaching effect for highly pigmented tooth substrates, more bleaching sessions might be necessary for the desired colour change.

In addition, the entire enamel surface was treated with the resin infiltration. In clinical scenarios, only selected enamel areas with WSL receive the infiltrating agent, and the bleaching procedure might reach the dental structures to a greater extent.^5,23^ There are to date no clinical studies that evaluate whether resin infiltration may act as a barrier like fixed appliances do, and possibly lead to decreased bleaching efficacy. Clinical studies should be performed to assess whether resin infiltration may prevent gel efficacy under treated surfaces. Also, bleaching treatment may be indicated prior to resin infiltration. Characteristics such as barrier thickness, size of the area of application, degree of tooth staining, and aging after resin infiltration with possible formation of porosities may influence the results. Furthermore, the use of hydrochloric acid might alter the colour of previously pigmented enamel, which could contribute to the bleaching results. The authors believe taking a color reading after the infiltration-protocol reading would further the understanding of the infiltrant procedure on the influence of color change before bleaching. More studies are needed to detect such an influence. Considering the limitations of laboratory studies, further investigations should be conducted to complement the present findings.

## Conclusion

Resin infiltration may act as a semipermeable barrier for carbamide peroxide gels. A positive bleaching response was observed for lower vs high tooth pigmentation.
